# Predicting the Water Sorption in ASDs

**DOI:** 10.3390/pharmaceutics14061181

**Published:** 2022-05-31

**Authors:** Dominik Borrmann, Andreas Danzer, Gabriele Sadowski

**Affiliations:** Laboratory of Thermodynamics, Department of Chemical and Biochemical Engineering, TU Dortmund University, Emil-Figge-Str. 70, 44227 Dortmund, Germany; dominik.borrmann@tu-dortmund.de (D.B.); andreas.danzer@tu-dortmund.de (A.D.)

**Keywords:** NET-GP, PC-SAFT, water-sorption isotherms, water-sorption kinetics, ASDs

## Abstract

Water decreases the stability of amorphous solid dispersions (ASDs) and water sorption is, therefore, unwanted during ASD storage. This work suggests a methodology to predict the water-sorption isotherms and the water-sorption kinetics in amorphous pharmaceutical formulations like ASDs. We verified the validity of the proposed methodology by measuring and predicting the water-sorption curves in ASD films of polyvinylpyrrolidone-based polymers and of indomethacin. This way, the extent and the rate of water sorption in ASDs were predicted for drug loads of 0.2 and 0.5 as well as in the humidity range from 0 to 0.9 RH at 25 °C. The water-sorption isotherms and the water-sorption kinetics in the ASDs were predicted only based on the water-sorption isotherms and water-sorption kinetics in the neat polymer on the one hand and in the neat active pharmaceutical ingredient (API) on the other hand. The accurate prediction of water-sorption isotherms was ensured by combining the Perturbed-Chain Statistical Association Theory (PC-SAFT) with the Non-Equilibrium Thermodynamics of Glassy Polymers (NET-GP) approach. Water-sorption kinetics were predicted using Maxwell–Stefan diffusion coefficients of water in the ASDs.

## 1. Introduction

Water sorption in pharmaceutical formulations may lead to unwanted phase transitions [1]. For instance, water may induce recrystallization of an API by both decreasing its solubility and by increasing its molecular mobility. Therefore, water sorption is a significant threat to the shelf life of amorphous solid dispersions (ASDs), where the API should remain amorphous and dissolved in the stabilizing polymer matrix. Consequently, predicting the water sorption in ASDs (i.e., water–sorption isotherms) is important for understanding the recrystallization of APIs in ASDs at humid conditions.

Early approaches used to predict water-sorption isotherms of ASDs assume that the water sorption in ASDs can be obtained as the weighted average of the water sorption in the polymer and in the API. Dalton et al. [2] investigated the validity of such a weighted-average approach for predicting the water-sorption isotherms of physical mixtures of pharmaceutical excipients. For that purpose, physical mixtures of pharmaceutical excipients with vastly different water uptakes (lactose, magnesium stearate, microcrystalline cellulose, starch, and hydroxyl propyl cellulose) were prepared. A weighted average of the water sorption of the individual excipients was appropriate for predicting the water-sorption isotherms of their respective amorphous mixtures. The reason for this is that the blending of the solid excipients did not result in a thermodynamic solution but rather physical mixtures of solid particles with almost no intermolecular interactions. Therefore, the ideal mixing behavior assumed by such a weighted-average approach might be a reasonable approach for these physical mixtures. However, ASDs behave very different from physical mixtures as the API is intentionally dissolved in the polymer and its solubility is caused by intermolecular interactions between the API and the polymer.

The Flory–Huggins model is often used to describe water-sorption isotherms of polymer-based solutions. However, Zhang and Zografi [3] reported difficulties when describing the water-sorption isotherms of PVP–sucrose and PVP–trehalose mixtures. The parts of the water-sorption isotherm where the mixture was glassy showed severe underestimations of the water uptake at sugar loads of the dry mixtures of 0, 0.25, and 0.5. Therefore, Zhang and Zografi [3,4] used the Flory–Huggins model in combination with the Vrentas model [5] to successfully model the water-sorption isotherms of PVP–sucrose, PVP–trehalose, and PVP–dextran mixtures. The Vrentas model requires measurements of the glass-transition temperatures (*T_g_*) and heat capacity differences of the PVP–sugar mixtures at the glass transition to model the water-sorption isotherms in these mixtures.

Crowley and Zografi [6] reported even more substantial discrepancies between a weighted-average approach and the measured water-sorption isotherms of ASDs. Water-sorption isotherms of PVP-based ASDs containing the APIs ursodeoxycholic acid, indapamide, or indomethacin (IND) were investigated. Water-sorption isotherms ranging from 0.1–0.8 drug load were modeled using the above mentioned combined Flory–Huggins/Vrentas model [3,4]. The descriptions of the water-sorption isotherms of PVP–IND ASDs for drug loads in the range of 0.1–0.4 showed excellent agreement with experimental data using constant binary interaction parameters between water/API, water/polymer and a drug load dependent interaction parameter between API/polymer. Furthermore, the authors reported strong deviations between measured and modeled water-sorption isotherms for ASDs with higher drug loads between 0.5 and 0.9. They explained these deviations proposing an immiscibility of PVP and IND. However, their hypothesis contradicted their dynamic scanning calorimetry measurements which suggested completely homogenous ASDs by showing only one *T_g_*.

Prudic et al. [7] used PC-SAFT to predict the water-sorption isotherms of PVP-naproxen ASDs. They also observed deviations between measured and predicted water-sorption isotherms which they explained by crystallization that co-occurred during the water-sorption measurements. This was supported by PXRD measurements. This explanation was further supported by the fact that the water uptake of these partially-crystallized ASDs was found in between the water uptakes predicted for the fully amorphous-ASDs and the fully-crystallized ASDs. However, the amount of crystalline material was not quantified.

The prediction of water-sorption isotherms of ASDs is difficult due to (1) the glassy nature of the ASD, (2) thermodynamic non-idealities, and (3) the superimposed thread of API crystallization. This work avoids crystallization during water sorption by investigating ASDs that are known to remain crystal free even after reaching a constant water uptake at certain conditions of relative humidity (RH) and temperature. PVP-based ASDs with IND are an excellent fit for this investigation, as verified by Prudic et al. [7] and Crowley and Zografi [6] due to the low recrystallization velocity of IND. 

Additionally, an often overlooked property is the water-sorption kinetics in ASDs. The water-sorption isotherm gives the water uptake after infinite time (in thermodynamic equilibrium). In contrast, the water-sorption kinetics determines the time to reach a certain water uptake at a given RH. Research on the water-sorption kinetics in ASDs is rare. Bunjes et al. [8] showed that the water-sorption kinetics in tablets made of microcrystalline cellulose and either eudragit, hydroxypropylmethylcellulose acetyl-succinate, or PVP–co-vinyl acetate (PVPVA) are quite different. However, a methodology to predict the water-sorption kinetics in ASDs has not been proposed yet. This work measured and predicted the water-sorption isotherms and water-sorption kinetics in PVP–IND and PVPVA–IND ASDs. First, the water-sorption isotherms were predicted via PC-SAFT combined with the NET-GP approach. Then, predicted Fickian diffusion coefficients and Maxwell–Stefan water-diffusion coefficients were compared to measured water-sorption kinetics. Finally, the water sorption in ASD was predicted using the predicted water-sorption isotherm and the predicted Maxwell–Stefan water-diffusion coefficients. 

## 2. Materials and Methods

### 2.1. Materials

Poly(vinylpyrrolidone) (PVP) with the grade K25 [CAS Nr. 9003-39-8] was purchased from Sigma-Aldrich with an average molar mass of $M_{p}=25,700\;g/\mathrm{mol}$. Copovidone (PVPVA64) (CAS Nr. 25086-89-9) with an average molar mass $M_{p}=65,000\;g/\mathrm{mol}$ was purchased by Dow Chemicals. Crystalline indomethacin (IND) was purchased from TCI (CAS-ID: 53-86-1). Ethanol with purity greater than 99.9% (LiChroSolv) was purchased from Merck. The water used for the sorption measurements was purified with a Millipore^®^ purification system (Merck, Darmstadt, Germany).

### 2.2. Film Preparation

We applied a spin-coating technique described in our previous study [9] to prepare PVP–IND and PVPVA–IND ASD films. A circular glass coverslip (18 mm in diameter) was masked with a perforated polystyrene foil and then coated with an ethanolic polymer-API solution using a spin-coating device (Süss MicroTec D80T2 spin coater). Ethanolic PVPVA–IND solutions used to create ASD films with a drug load of 0.2 contained 35 wt% ethanol and were spun at 2200 rpm. The ethanolic PVPVA–IND solutions used to create ASD films with a drug load of 0.5 containing 22 wt% ethanol were spun at 1100 rpm. The PVP–IND solutions contained 58 wt% ethanol for both drug loads and were spun at 2000 rpm for the drug load of 0.2 and at 1150 rpm for a drug load of 0.5. These specifications allowed the preparation of ASD films with thicknesses of ~7–9 µm, making them comparable to the PVP and PVPVA films and the amorphous IND films from our previous works [9,10]. Furthermore, the geometry of all considered films represents an ideal cylinder, and all films share the same circular base area with a diameter of 14.5 mm. 

### 2.3. Water-Sorption Measurements

The mass gain of the ASD films was measured at 25 °C via a dynamic vapor sorption (DVS) device (DVS Intrinsic Plus). The device from Surface Measurements Systems has a built-in balance with a precision of 0.1 µg. The ASD films were dried in the measurement cell at an RH of 10^−5^ for at least 12 h to obtain the dry mass $m_{0}$ of the ASD film. Then, six successive step-wise changes in the range from 0 to 0.9 RH were investigated. Thus, a RH step represents an immediate increase from the previous RH to a new RH where it was held constant until a next RH step was applied. The duration of each RH step was terminated automatically applying a mass-change-rate criterium < 0.0001 wt%/min (sorption rate 1 µg/g/min). The mass of water $m_{w}$ resulted as the difference between the readings of the total mass m and the dry mass $m_{0}$ of the ASD. Water weight fractions $w_{w}$ were then accessible as the ratio of the mass of water $m_{w}$ and the total mass m. The mass m was measured as function of time and plotting the water weight fractions $w_{w}$ versus time resulted in the water-sorption curve. The endpoints of the water-sorption curves resulting from the RH steps denote points of the water-sorption isotherm whereas parts of the water-sorption curve between startpoint and endpoint determine the water-sorption kinetics. Water-sorption measurements were performed in triplicates and average values were reported with their standard deviations.

### 2.4. Crystallinity Check

**Figure 1:**
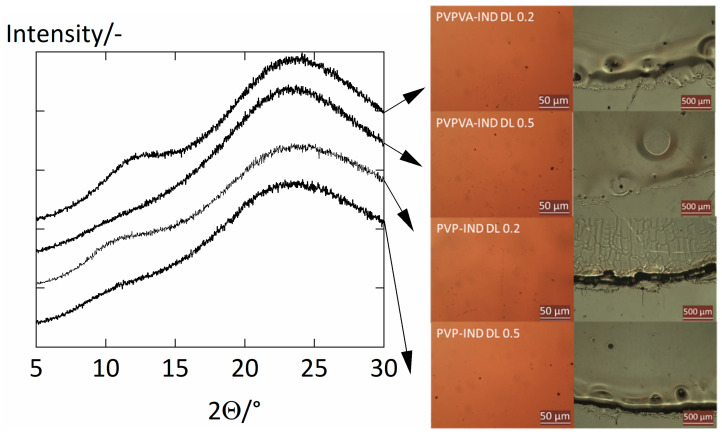
PXRD measurements (**left**) of amorphous PVP–IND ASD films and PVPVA–IND ASD films with drug loads 0.2 and 0.5 after the water-sorption measurements. In addition, optical microscope images (**right**) were taken after the water-sorption measurements.

After the water-sorption measurements, we performed powder X-ray diffraction (PXRD) measurements to verify the amorphousness of the ASDs after the sorption experiments. The device used in this work was a PXRD Mini Flex 600 from Rigaku. The ASD films were irradiated using a Cu Kα irradiation source with a voltage of 40 kV and a current of 15 mA. The samples were investigated in a step-scan mode in the region of 5 < 2Θ < 30°, where Θ is the angle of the detector with a step size of 5°/min. Additionally, we used an optical microscope (Leica DM4000M) with a polarization filter and a magnification level of 50 times and 5 times to further support the amorphousness of the ASD films. The absence of any characteristic peaks of crystalline IND (2Θ = 11.8° and 2Θ = 22°) [11] in the PXRD results (Figure 1) suggests that the crystal mass was below the detection limit of the PXRD device. Furthermore, the optical microscopy images agree with this conclusion.

## 3. Modeling

### 3.1. PC-SAFT

The reduced residual Helmholtz energy $a^{res}$ was described using Perturbed-Chain Statistical Associating Fluid Theory (PC-SAFT) as shown in Equation (1).

(1) $$a^{res}=a^{hc}+a^{disp}+a^{assoc}$$

$a^{hc}$ is the reduced Helmholtz energy contribution for a hard-chain fluid, $a^{disp}$ is the dispersion contribution, and $a^{assoc}$ the association contribution to the reduced Helmholtz energy [12]. PC-SAFT uses the segment number $m_{i}^{Seg}$, segment diameter $\sigma_{i}$, dispersion energy parameter $u_{i}/k_{B}$, the association-energy parameter $\varepsilon^{AiBi}/k_{B}$, and association volume $\kappa^{AiBi}$ to characterize a component i ($k_{B}$ is the Boltzmann constant). Berthelot-Lorenz mixing rules were applied to calculate the segment diameter $\sigma_{ij}=\frac{\sigma_{i}+\sigma_{j}}{2}$ and the dispersion energy parameter $u_{ij}/k_{B}$ (Equation (2)) of a binary pair of components i and j.

(2) $$u_{ij\;}=\sqrt{u_{i}u_{j}}\left(1-k_{ij}\right)$$

Here, $u_{i}$ is the dispersion energy of component i and $u_{ij}$ is the dispersion energy in the mixture. The binary interaction parameter $k_{ij}$ is introduced to correct the geometric mixing rule for the dispersion energy $u_{ij}$.

For the association-energy parameter $\varepsilon^{AiBj}$ and the association volume $\kappa^{AiBj}$ in the mixture, mixing rules of Wolbach and Sandler [13] were applied.

The fugacity $f_{i}$ of a component i was calculated according to Equation (3).

(3) $$\ln\left(f_{i}\right)=a^{res}+{\left(\frac{\partial a^{res}}{\partial x_{i}}\right)}_{T,\tilde{\rho },x_{j\neq i}}-\underset{j}{\sum }x_{j}{\left(\frac{\partial a^{res}}{\partial x_{j}}\right)}_{T,\tilde{\rho },x_{j\neq i}}+Z-1+\ln\left(\tilde{\rho }x_{i}k_{B}T\right)$$

Here, $x_{i}$ is the mole fraction of component i, $\tilde{\rho }$ is the number density, T is the system temperature. The compressibility factor Z was obtained according to Equation (4)

(4) $$Z=1+\tilde{\rho }{\left(\frac{\partial a^{res}}{\partial \tilde{\rho }}\right)}_{T,x_{i}}$$

### 3.2. Calculations of Water-Sorption Isotherms

Due to the reduced mobility of the molecules, a glassy ASD is in a pseudo-equilibrium with the surrounding vapor phase, whereas rubbery ASDs are in equilibrium with the latter. The water-sorption isotherms of both glassy and rubbery ASDs were calculated using the isofugacity criterion in Equation (5).

(5) $$f_{w}^{L}=f_{w}^{V}\left(p,T\right)$$

Here, $f_{w}^{L}$ is the fugacity of water in the ASD–water mixture and $f_{w}^{V}$ is the fugacity of water in the vapor phase surrounding the ASD. The total pressure p was equal to the partial pressure of water $p_{w}=p_{0w}^{LV}\;RH$ where $p_{0w}^{LV}$ is the vapor pressure of water and RH is the relative humidity. The fugacity of water $f_{w}^{L}$ in the ASD–water mixture was described using PC-SAFT with and without the NET-GP approach [14] for glassy and rubbery ASDs, respectively. A rubbery ASD–water mixture is in an equilibrium state (EQ) with the volume $V^{EQ}$ of the ASD–water mixture. In contrast, a glassy ASD–water mixture has a non-equilibrium (NE) volume $V^{NE}$ due to the kinetically hindered molecular mobility. The volume of a system in equilibrium was modeled at system temperature and pressure using PC-SAFT. However, substantial deviations occur when modelling the volume of glassy mixtures. The NET-GP approach was used together with PC-SAFT providing an accurate representation of the pressure–volume–temperature behavior of a glassy polymer [9]. We applied the transition rule for the fugacity of water $f_{w}^{L}$ displayed in Equation (6) derived in our previous work [9].

(6) $$f_{w}^{L}=\{\begin{array}{c} f_{w}^{L}\left(T,p,V^{EQ}\left(T,p\right),x_{i}\right)\;if\;x_{w}^{EQ}>x_{w}^{NE}\\ f_{w}^{L}\left(T,p,V^{NE},x_{i}\right)\;if\;x_{w}^{EQ}\leq x_{w}^{NE} \end{array}$$

The PC-SAFT modeling with NET-GP switches to PC-SAFT modeling without NET-GP when the equilibrium mole fraction $x_{w}^{EQ}$ of water becomes greater than the non-equilibrium mole fraction $x_{w}^{NE}$ of water. PC-SAFT modeling without NET-GP requires the volume $V^{EQ}$ of the ASD–water mixture in equilibrium at the system’s temperature T and pressure p. In contrast, PC-SAFT modeling with NET-GP requires the volume $V^{NE}$ of the ASD–water mixture in non-equilibrium given by Equation (7) as a function of the relative humidity RH as used in previous work [9].

(7) $$\frac{V_{0}^{NE}}{V^{NE}}=\frac{v_{0}^{NE}}{v^{NE}}\left(1-w_{w}\right)=1-k_{w}^{NE}RH^{2}$$

The ratio of the volume $V^{NE}$ of the ASD–water mixture in non-equilibrium and the volume $V_{0}^{NE}$ of the dry ASD in non-equilibrium was expressed in terms of the specific volume $v^{NE}$ of the ASD–water mixture, the specific volume $v_{0}^{NE}$ of the dry ASD in non-equilibrium and the water weight fraction $w_{w}$. Furthermore, $k_{w}^{NE}$ is the swelling coefficient of the ASD caused by water. It was assumed, that the specific volume $v_{0}^{NE}$ of the dry ASD in non-equilibrium is the weighted average of the specific volumes $v_{0p}^{NE}$ of the polymer and $v_{0a}^{NE}$ of the API in non-equilibrium (Equation (8)).

(8) $$v_{0}^{NE}=w_{0a}v_{0a}^{NE}+w_{0p}v_{0p}^{NE}$$

The quantity $w_{0a}$ represents the drug load and the quantity $w_{0p}=\left(1-w_{0a}\right)$ represents the polymer load of the dry ASD. The NET-GP approach for polymer blends, as proposed by Sarti and Dogheri [15], suggests a volumetric mixing rule for $k_{w}^{NE}$. Hence, the swelling coefficient $k_{w}^{NE}$ of the ASD by water was described according to Equation (9).

(9) $$k_{w}^{NE}=w_{0a}\frac{v_{0a}^{NE}}{v_{0}^{NE}}k_{wa}^{NE}+w_{0p}\frac{v_{0p}^{NE}}{v_{0}^{NE}}k_{wp}^{NE}\;$$

Here, $k_{wp}^{NE}$ is the swelling coefficient of the polymer by water and $k_{wa}^{NE}$ is the swelling coefficient of the API by water. The parameters $v_{0i}^{NE}$ and $k_{wi}^{NE}$ are displayed in Table 1.

### 3.3. Water-Sorption Kinetics

The Cranc equation [16] (Equation (10)) models the solvent diffusion in a film of thickness $L_{0}$ and was used in this work to fit and predict the water-sorption kinetics in the ASDs.

(10) $$m_{w}=\left(m_{w}^{\infty }-m_{w}^{0}\right)\left(1-\overset{\infty }{\underset{q=0}{\sum }}\frac{8}{\pi^{2}{\left(2+q\right)}^{2}}\exp\left({\left(2+q\right)}^{2}\frac{D_{w}}{4L_{0}^{2}}\;t\right)\right)+m_{w}^{0}$$

Here, $m_{w}^{0}$ and $m_{w}^{\infty }$ are the water masses corresponding to start and end of the sorption steps. q is the index to approximate the infinite series and 20 summands turned out to be sufficient. It is assumed that water is the only mobile species. This way, the Fickian water-diffusion coefficient $D_{w}$ in the ASD was expressed in terms of Equations (11) and (12).

(11) $$D_{w}=\omega_{0}^{2}\Gamma_{w}^{\prime \prime }{Ð}_{w}^{\prime \prime }$$

(12) $$\Gamma_{w}^{\prime \prime }=\frac{\partial \ln f_{w}^{L}}{\partial \ln w_{w}}$$

The non-idealities in the diffusion were corrected by applying the thermodynamic factor of water $\Gamma_{w}^{\prime \prime }$ which leads to the segmental Maxwell–Stefan diffusion coefficient ${Ð}_{w}^{\prime \prime }$ for water in the ASD. Moreover, during water sorption, the ASD volume substantially increases, and the reference frame needs to be fixed. This was achieved using a mass-fixed reference frame (see Cranc [16]) which results in an effective flux reduction by the factor $\omega_{0}^{2}={\left(\frac{L\;}{L_{0}}\right)}^{2}$ correcting for the growing discrepancy between the actual thickness L and the thickness of the dry ASD film $L_{0}$. The average thicknesses $L_{0}$ of the dry films were calculated from their dry mass $m_{0}$, the densities of the dry ASD films (estimated using the pure densities $\rho_{oi}$ in Table 1) and their cross-sectional area. The time-dependent thickness L of the films was estimated assuming volume additivity and using the density $\rho_{ow}$ of water. 

The segmental Maxwell–Stefan water diffusion ${Ð}_{w}^{\prime \prime }$ in the ASD was predicted using Equation (13) the derivation of which can be found in the Supplementary Material.

(13) $${Ð}_{w}^{\prime \prime }={\left(\frac{w_{0a}}{{Ð}_{wa}^{\prime \prime }\;}+\frac{w_{0p}}{{Ð}_{wp}^{\prime \prime }}\right)}^{-1}$$

${Ð}_{wa}^{\prime \prime }$ is the segmental Maxwell–Stefan diffusion coefficient of water in the API and ${Ð}_{wp}^{\prime \prime }$ is the segmental Maxwell–Stefan diffusion coefficient of water in the polymer. 

### 3.4. Water Concentration Dependency of the Water-Diffusion Coefficients

We utilized concepts from the free-volume theory to predict the water-concentration dependency of ${Ð}_{w}^{\prime \prime }$ using the water concentration dependencies of ${Ð}_{wa}^{\prime \prime }$ and ${Ð}_{wp}^{\prime \prime }$. As demonstrated in previous studies [10,11], the free-volume theory can be used to describe the water-concentration dependencies of ${Ð}_{wa}^{\prime \prime }$ in amorphous APIs, and of ${Ð}_{wp}^{\prime \prime }$ in PVP-based polymers. The central assumption of the free-volume theory is that solvent(water) diffusion coefficients depend on the free volume of the system, which is the volume that is not occupied by the molecules of that system. 

**Figure 2:**
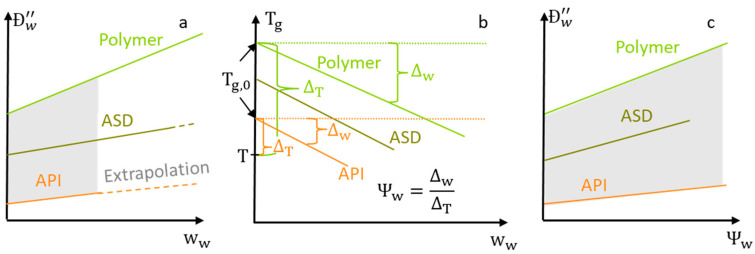
Schematic of the water-diffusion coefficients in a hydrophilic polymer, an API, and in their ASD at constant temperature. (**a**) shows the water-diffusion coefficients as a function of the water weight fractions $w_{w}$. Experimentally inaccessible values that would require extrapolation are indicated as dashed lines. (**b**) *T_g_* reduction by water as used to calculate the plasticization factor $\Psi_{w}$. Diagram (**c**) shows the same water-diffusion coefficients as (**a**) a function of the plasticization factor $\Psi_{w}$. Regions that are accessible via interpolation are shown as grey boxes in (**a**,**c**).

Sturm et al. [17] proposed an extension to the free-volume theory that applies to glassy and rubbery polymer–solvent mixtures. This extension states that the free volume of a glassy polymer-solvent mixture depends (1) on the distance $\Delta_{T}=T_{g,0}-T$ of the glass transition temperature $T_{g,0}$ of the dry system to the system temperature *T* and (2) on the distance $\Delta_{w}=T_{g,0}-T_{g}$ of the glass transition temperature $T_{g,0}$ of the dry system to the glass transition temperature $T_{g}$ of the solvent(water)-loaded system (see Figure 2b). In this work, we propose the plasticization factor $\Psi_{w}$ (Equation (14)) that combines the two contributions into one variable.

(14) $$\Psi_{w}=\frac{\Delta_{w}}{\Delta_{T}}=\frac{T_{g,0}-T_{g}}{T_{g,0}-T}$$

Expressing the water concentration dependency of ${Ð}_{w}^{\prime \prime }$ in terms of the plasticization factor $\Psi_{w}$ has significant advantages over using the water weight fraction $w_{w}$ for that purpose. This is because the API’s water uptake is significantly lower than the ASD’s water uptake when the ASD is based on a hydrophilic polymer. As a result, the experimental determination of ${Ð}_{wa}^{\prime \prime }$ via water-sorption measurements is impossible at the same water weight fractions as for the ASD. Thus, any function describing the water concentration dependency of ${Ð}_{wa}^{\prime \prime }$ in terms of $w_{w}$ would require an extensive extrapolation to the higher water weight fractions $w_{w}$ in the ASD (Figure 2a).

The higher water uptake polymer compared to the one of the API (resulting in a higher $\Delta_{w}$) is compensated by the polymer’s higher $T_{g,0}$ (resulting in a higher $\Delta_{T}$) (see Figure 2b). As a result, the plasticization factors $\Psi_{w}$ for most ASDs, polymers and APIs are similar. As a consequence, we use a piecewise-linear polynomial of the experimentally determined water concentration dependency of ${Ð}_{wa}^{\prime \prime }\left(\Psi_{w}\right)$ and ${Ð}_{wp}^{\prime \prime }\left(\Psi_{w}\right)$ from previous work [10,11] (summarized in Table 2) for safe interpolations when predicting ${Ð}_{w}^{\prime \prime }\left(\Psi_{w}\right)$ in this study (see Figure 2c).

The glass transition temperature $T_{g}$ was predicted using the Gordon-Taylor equation [18] (Equation (15)).

(15) $$T_{g}=\frac{K_{a}w_{a}T_{g0a}+K_{p}w_{p}T_{g0p}+w_{w}T_{g0w}}{K_{a}w_{a}+K_{p}w_{p}+w_{w}}$$

Here, $K_{a}$ and $K_{p}$ are the Gordon-Taylor constants of the API-water and polymer-water mixtures and $T_{g0a}$ and $T_{g0p}$ are the glass-transition temperatures of the API and the polymer, respectively. 

${Ð}_{w}^{\prime \prime }$,$\;{Ð}_{wa}^{\prime \prime }$, and $\;{Ð}_{wp}^{\prime \prime }$ were assumed to be constant during a sorption step and the water-concentration dependent quantities $\Gamma_{w}^{\prime \prime }$, $\omega_{0}^{2}$, $T_{g}$,${\;\mathrm{and}\;\Psi }_{w}$ were evaluated at an intermediate water weight fraction of $0.3w_{w}^{0}+0.7w_{w}^{\infty }$ as proposed by Vrentas et al. [19]. Here, $w_{w}^{0}$ is the water weight fraction at the start and $w_{w}^{\infty }$ is the water weight fraction at the end of the sorption step.

### 3.5. Model Parameters

PC-SAFT pure component parameters for PVP, PVPVA, and IND, binary interaction parameters of the components with water and their NET-GP parameters are summarized in Table 1. The binary interaction parameter $k_{ij}=-0.0118\;T+0.0922$ between PVP and IND was determined by Prudic et al. [20]. The binary interaction parameter $k_{ij}=-0.0621$ between PVPVA and IND was fitted in this study to the solubilities of IND in PVPVA which is shown in Figure S1 in the Supporting Information.

**Table 1:** Association sites $N_{i}$ and the other PC-SAFT and NET-GP parameters as well as pure densities $\;\rho_{0i}$ of the components considered in this work. Parameters which are not needed are marked as not available (N.A.).

	PVP [7]	PVPVA [21]	IND [22]	Water [23]
$M_{i}/\frac{g}{mol}$	25,700	65,000	357.79	18.02
$m_{i}^{Seg}/M_{i}/\frac{mol}{g}$	0.0407	0.0372	0.03992	0.06687
$\sigma_{i}/Å$	2.71	2.947	3.535	2.7971
$u_{i}/k_{B}/\;K$	205.992	205.271	262.791	353.94
$\epsilon^{AiBi}/k_{B}/\;K$	0	0	886.44	2425.67
$\kappa^{AiBi}/-$	0.02	0.02	0.02	0.0451
$N_{i}/-$	231/231	653/653	3/3	1/1
$k_{wi}/-$	−0.128 ^a^	−0.128 ^a^	−0.022 ^b^	N.A.
$v_{0i}^{NE}/\frac{cm^{3}}{g}\;$	0.6637 ^a^	0.7478 ^a^	$v_{0a}^{EQ}$	N.A.
$k_{wi}^{NE}/-$	0.4279 ^a^	0.244 ^a^	0	N.A.
$\rho_{0i}/\frac{kg}{m^{3}}$	1250	1190	1320	997
$T_{g0i}/K$	441.51	383.9	317.6	136
$K_{i}/-$	0.253 ^c^	0.3 ^c^	0.11 [24]	N.A.

^a^ taken from previous work [9], ^b^ taken from previous work [10], ^c^ estimated using the Simha–Boyer rule [25] using $\;\rho_{0i}$ and $T_{g,0i}$.

The specific volume $v_{0a}^{NE}$ of IND in non-equilibrium was assumed to be identical to the specific volume of $v_{0a}^{EQ}$ in equilibrium calculated by PC-SAFT without NET-GP and the non-equilibrium parameter between water and API was set to zero ($k_{wa}^{NE}=0$).

The water-concentration dependency of ${Ð}_{wa}^{\prime \prime }$ for water in IND was calculated from a previous work [10] which determined the Maxwell–Stefan diffusion coefficient ${Ð}_{\mathrm{wa}}$ of water in IND. ${Ð}_{\mathrm{wa}}$ considers molecular friction instead of segmental friction. Converting the two diffusion coefficients was done via ${Ð}_{wa}^{\prime \prime }={Ð}_{wa}\frac{1-x_{w}}{1-w_{w}}$. Then, for each pair of ${Ð}_{wa}^{\prime \prime }$ and its corresponding $w_{w}$ from this previous work [10], a plasticization factor $\Psi_{w}$ was calculated via Equation (14). The water concentration dependencies of ${Ð}_{wp}^{\prime \prime }$ for water in PVPVA or PVP were already determined in an another work [9] and the corresponding plasticization factors $\Psi_{w}$ were calculated in the same manner. The pairs of $\Psi_{w}$ and the corresponding values for ${Ð}_{wa}^{\prime \prime }$ and ${Ð}_{wp}^{\prime \prime }$ are listed in Table 2. Finally, a piecewise-linear interpolation of these pairs serves as a function relation of ${Ð}_{wa}^{\prime \prime }\left(\Psi_{w}\right)$ and ${Ð}_{wp}^{\prime \prime }\left(\Psi_{w}\right)$ used for the prediction of ${Ð}_{w}^{\prime \prime }\left(\Psi_{w}\right)$ via Equation (13).

**Table 2:** Segmental Maxwell–Stefan diffusion coefficients ${Ð}_{wa}^{\prime \prime }$ of water in IND and ${Ð}_{wp}^{\prime \prime }$ of water in PVP as well as in PVPVA as a function of the plasticization factor $\Psi_{w}$ at 25 °C.

	PVP ^a^		PVPVA ^a^		IND ^b^	
$RH\;/10^{-2}$	$\Psi_{w}$ /-	${Ð}_{wp}^{\prime \prime }{\;}^{a}$ /10^−15^ m^2^ s^−1^	$\Psi_{w}$ /-	${Ð}_{wp}^{\prime \prime }{\;}^{a}$ /10^−15^ m^2^ s^−1^	$\Psi_{w}$ /-	${Ð}_{wa}^{\prime \prime }{\;}^{b}$ /10^−15^ m^2^ s^−1^
9.24	0.14671	255.4	0.07363	340.7	0.05414	44.8648
29.4	0.453	94.7	0.27951	301.5	0.2415	18.5876
44.5	0.70977	57.8	0.50773	75.9	0.48449	22.2132
59.9	0.91512	12.4	0.74883	106.2	0.777	58.6949
73.4	1.10884	223.6	1.01617	184.3	1.12032	125.916
87.8	1.34422	345.4	1.40619	610.6	1.58168	166.599

^a^ Taken from previous work [9] ^b^ calculated from previous work [10].

## 4. Results and Discussion

### 4.1. Prediction of Water-Sorption Isotherms

**Figure 3:**
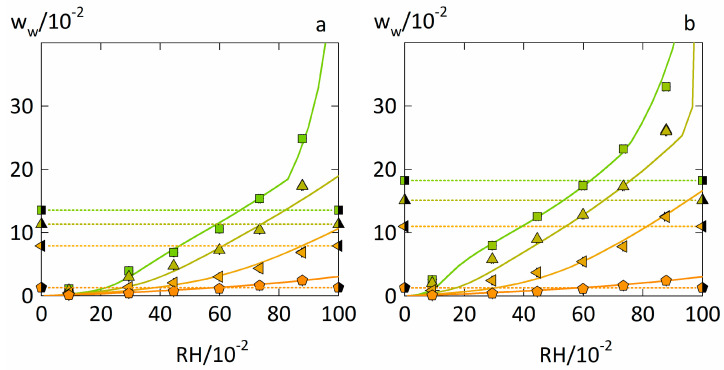
Water-sorption isotherms of PVPVA–IND ASDs (**a**) and PVP–IND ASDs (**b**) at 25 °C. Up-side triangles and left-side triangles indicate ASDs with 0.2 drug load and 0.5 drug load, respectively. Water-sorption isotherms for PVP and PVPVA (squares) and for amorphous IND (pentagons) were taken from previous works [9,10]. Predicted water weight fraction that result in a *T_g_* of 25 °C (Equation (15)) are displayed as dotted horizontal lines and connected via half-filled versions of the same symbols as used for the corresponding water-sorption isotherms. Solid lines represent the water-sorption isotherms predicted using PC-SAFT and NET-GP.

The measured and predicted water-sorption isotherms (Equation (5)) in PVPVA–IND and PVP–IND ASDs are displayed in Figure 3. Overall, the water uptake in the two ASD–water systems with drug load of 0.5 is roughly only one third compared to the one in the polymers PVP and PVPVA at the same RH. Thus, the presence of IND reduces the water uptake in these ASDs significantly. Just based on the water-sorption isotherms in the pure components PVP, PVPVA, and IND, PC-SAFT combined with NET-GP almost quantitatively predicts the water-sorption isotherms of the PVP–IND and PVPVA–IND ASDs for both drug loads. 

The predicted water weight fractions at which the glass-transition temperatures *T_g_* of the ASD–water systems reach 25 °C suggest that the ASDs with a drug load of 0.5 remain glassy until 0.85 RH for the PVP–IND ASD and 0.9 RH for the PVPVA–IND ASD. In contrast, the corresponding polymers are glassy only until 0.6 RH for PVP and 0.65 RH for PVPVA. Although IND reduces the glass transition temperature of the ASDs when dry, its presence also decreases the water uptake of the ASDs significantly. The latter effect of IND is obviously more substantial than its reducing effect on the glass transition temperature of the dry ASD. As a result, these PVP-based ASDs remain glassy for higher RHs. 

### 4.2. Experimental Water-Sorption Curves

**Figure 4:**
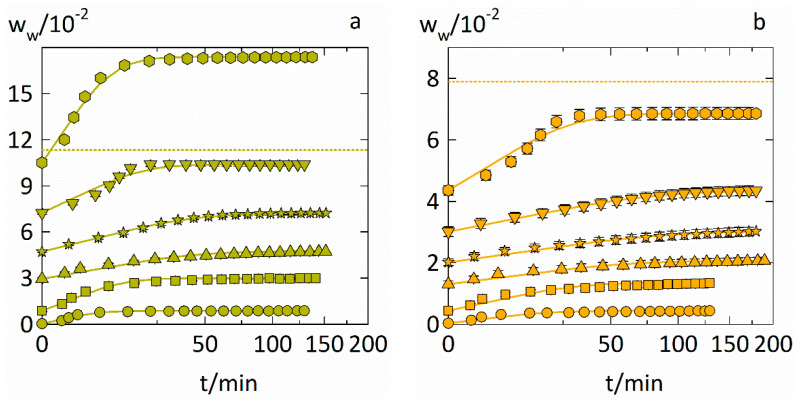
Water-sorption curves in PVPVA–IND ASD at T = 25 °C. The evolution of the water mass fractions in ASDs with drug loads of 0.2 (**a**) and 0.5 (**b**) are displayed for six RH step changes. Each step change is displayed via different symbols (circles: 0 to 0.1 RH, squares: 0.1 to 0.3 RH, up-side triangles: 0.3 to 0.45 RH, stars: 0.45 to 0.6 RH, down-side triangles: 0.6 to 0.75 RH, hexagons: 0.75 to 0.9 RH, where these RHs are rounded and the exact RHs of the six sorption steps are displayed in Table 3) while the fittings with Equation (10) are indicated as solid lines. Predictions of the water-weight fraction that result in a *T_g_* of 25 °C by Equation (15) are displayed as dotted horizontal lines.

The water-sorption curves of PVPVA–IND ASD with drug loads of 0.2 and 0.5 are displayed in Figure 4. Fickian water-diffusion coefficients $D_{w}$ in the ASD were determined from these curves by fitting the data using Equation (10). The resulting water-diffusion coefficients are displayed in Table 3. The fittings to the water-sorption curves for PVP–IND ASD are shown in the Figure S2 in Supporting Information.

As the time axes are scaled as square root of time, anomalous sorption kinetics appears when the slope of the curves is non-linear for the first 60% of the total water uptake. Anomalous sorption behavior appears when solvent diffusion in a system is altered by a slow relaxation of a polymer [26]. The water-sorption curve from 0.6 to 0.75 RH in the ASD containing a drug load of 0.2 (Figure 4a) shows a strong sigmoidal curvature indicating anomalous sorption behavior. The ASD containing a drug load of 0.5 shows a similar sigmoidal curvature at the sorption-step from 0.75 to 0.9 RH and with a more pronounced upwards curvature compared to the ASD containing a drug load of 0.2. The fittings of Equation (10) did not reproduce these details of the sigmoidal sorption curves. Such sigmoidal sorption curves were already observed in our previous work [9] for the water-sorption curves in PVP and PVPVA in the vicinity of their glass transition. Therefore, it is reasonable that ASDs based on these polymers also show this anomalous sorption behavior in the vicinity of their glass transition. The sharp upwards curvature of these sorption curves indicates a strong acceleration of the water diffusion, likely caused by a substantial increase in molecular mobility. This accelerated sorption behavior was already used as an indicator for the glass transition as demonstrated by Dohrn et al. [27] using a RH-ramping method in the DVS instead of discrete RH steps. Consequently, the predictions of the glass transition by Equation (15) qualitatively predict the true glass transition of these PVPVA–IND ASDs.

For water-sorption curves that behave anomalous, the physical meaningfulness of the determined Fickian water-diffusion coefficients (Table 3) might be limited, as the water sorption in those cases is not only controlled by water diffusion but also by and polymer volume relaxation [26]. Despite that, the fittings do qualitatively capture the time constants of the water-sorption kinetics.

### 4.3. Prediction of Water-Diffusion Coefficients in ASDs

**Figure 5:**
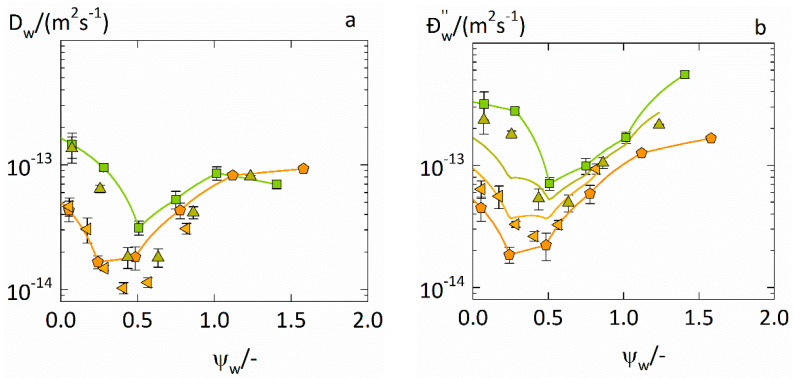
Water-diffusion coefficients in PVPVA–IND ASDs at 25 °C. Fickian water-diffusion coefficients $D_{w}\;$ in ASDs (**a**) from fittings of Equation (10) and the corresponding segmental Maxwell–Stefan diffusion coefficients ${Ð}_{w}^{\prime \prime }$ in ASDs (**b**) via Equation (11) are displayed as up-side triangles (for a drug load of 0.2) and left-side triangles (drug load of 0.5), respectively. The Fickian diffusion coefficients $D_{wa}$ of water in IND (pentagons) and $D_{wp}$ of water in PVPVA (squares) were taken from previous works [9,10]. Additionally, the predicted Maxwell–Stefan diffusion coefficients ${Ð}_{w}^{\prime \prime }$ of water in the ASDs are displayed as solid lines.

Segmental Maxwell–Stefan diffusion coefficients ${Ð}_{w}^{\prime \prime }$ were calculated from the experimentally determined Fickian diffusion coefficients $D_{w}$ in PVPVA–IND ASDs from Table 3 via Equation (11) and are displayed in Figure 5. These ${Ð}_{w}^{\prime \prime }$ values are compared to the predicted ${Ð}_{w}^{\prime \prime }$ based on Equation (13) which is also displayed in Figure 5. The same comparison for ${Ð}_{w}^{\prime \prime }$ and $D_{w}$ in PVP–IND ASDs is shown in Figure S3 of the Supporting Information.

The experimentally determined Fickian diffusion coefficients $D_{w}$ in the ASD (Figure 5a) and the corresponding Maxwell–Stefan diffusion coefficients ${Ð}_{w}^{\prime \prime }$ in the ASD calculated (Figure 5b) decrease with increasing plasticization factor, reach minima, and then rise again. Thus, both $D_{w}$ and ${Ð}_{w}^{\prime \prime }$ show non-monotonous water-concentration dependencies for both drug loads. The non-monotonous water-concentration dependencies of the water-diffusion coefficients in IND and in PVPVA were already explained in our previous works [9,10] and are the result of two counteractive effects on the free volume when transitioning from the glassy to the rubbery state. 

Further, as to be seen in Figure 5a, the experimentally determined Fickian diffusion coefficients $D_{w}$ in the ASD containing 0.5 drug load become even lower than the lowest value of the Fickian diffusion coefficients $D_{wa}$ of water in IND. In contrast, Figure 5b shows that the Maxwell–Stefan diffusion coefficients ${Ð}_{w}^{\prime \prime }$ are always higher than the lowest value of the Maxwell–Stefan diffusion coefficients ${Ð}_{wa}^{\prime \prime }$ of water in IND. This means that the apparent water diffusion in this ASD becomes even slower than in IND while the “true” water diffusion in this ASD lies in between the “true” water diffusion in both polymer and IND. $D_{w}$ just maps the apparent water diffusion in the ASD disregarding intermolecular interactions, whereas ${Ð}_{w}^{\prime \prime }$ explicitly considers the influence of these interactions leading to the “true” water diffusion in the ASD. This means that the intermolecular interactions in the considered systems deaccelerate the water diffusion in the ASDs which is correctly predicted by Equation (13). 

As to be seen from Figure 5b, Equation (13) also correctly predicts the non-monotonous water concentration dependencies of ${Ð}_{w}^{\prime \prime }$ for both drug loads solely based on the water concentration dependencies of ${Ð}_{wa}^{\prime \prime }$ and ${Ð}_{wp}^{\prime \prime }$ and can thus be used to predict the Maxwell–Stefan diffusion coefficients and the Fickian diffusion coefficients of water in the ASD. This way, the water-sorption curves in the ASDs can be fully predicted.

### 4.4. Prediction of the Water-Sorption Curves

**Figure 6:**
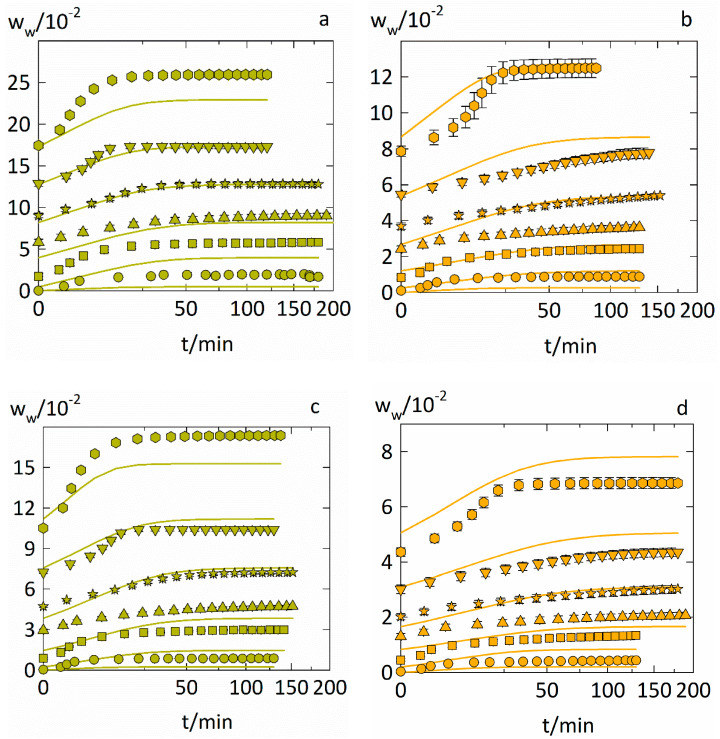
Prediction of the water-sorption curves in PVP–IND ASDs (**a**,**b**) and PVPVA–IND ASDs (**c**,**d**). The evolution of the water content (mass fractions) in ASDs with drug load of 0.2 (**a**,**c**) and drug load of 0.5 (**b**,**d**) are displayed for six step changes at T = 25 °C. Each step change is displayed via different symbols (circles: 0 to 0.1 RH, squares: 0.1 to 0.3 RH, up-side triangles: 0.3 to 0.45 RH, stars: 0.45 to 0.6 RH, down-side triangles: 0.6 to 0.75 RH, hexagons: 0.75 to 0.9 RH, where these RHs are rounded and the exact RHs of the six sorption steps are displayed in Table 3). Predictions from Equation (10) are indicated as solid lines.

We demonstrated that the water-sorption isotherms as well as the water-diffusion coefficients in ASDs can be predicted. This section now shows predictions of the entire water-sorption measurements in the ASDs. Thus, the start points and end points of the sorption steps were predicted via Equation (5) and used with the water-sorption kinetics (Equation (10)) that was supplied with $D_{w}$ predicted via Equation (13) using Equation (11). The predictions of water-sorption kinetics in PVP–IND ASDs and PVPVA–IND ASDs for drug loads of 0.2 and 0.5 are displayed in Figure 6.

Combining PC-SAFT and NET-GP with the Maxwell–Stefan formalism gives a reasonable overall prediction of the entire water-sorption measurement. Most significant deviations between measurement and predictions are caused by inaccuracies in predicting the starting and endpoints correctly. It becomes evident that deviations in the water-sorption isotherms (Equation (5)) are more decisive than accurately describing the water-diffusion coefficient’s water-concentration dependency.

## 5. Conclusions

This study gave insight into the water-sorption behavior of polyvinylpyrrolidone-based ASDs. The water-sorption isotherms and water-sorption kinetics in PVP–IND and PVPVA–IND ASD films with drug loads of 0.2 and 0.5 were measured, correlated, and predicted. 

PC-SAFT was used to predict the water-sorption isotherms in glassy and rubbery ASDs. Due to the significant reduction of the water uptake of these ASDs compared to the one in the pure polymers, the ASDs were glassy over a broader RH range than the corresponding polymers. As a result, we combined PC-SAFT with the NET-GP approach and the predictions showed excellent agreement with the experimental data. 

Fickian water-diffusion coefficients fitted from the water-sorption curves suggested that the water-sorption kinetics in the ASDs were slower than in both the polymers and IND. This phenomenon was explained via intermolecular molecular interactions, as the corresponding Maxwell–Stefan diffusion coefficients did not show such behavior. 

Finally, instead of just correlating measured water-sorption curves in ASDs, the water-diffusion coefficients in the ASD can be predicted just based on water-diffusion coefficients determined in the polymer on the one hand and in the API on the other hand. As a result, entire water-sorption curves in ASDs can be predicted. These predictions required no sorption data about the ASD–water systems.

## Figures and Tables

**Table 3:** RH, water-weight fraction $w_{w}^{\infty }$ at the end of the sorption step, and experimentally determined Fickian diffusion coefficients $D_{w}$ for PVP–IND and PVPVA–IND ASDs.

	PVPVA–IND ASDs				PVP–IND ASDs			
DL	0.2		0.5		0.2		0.5	
RH /10^−2^	$w_{w}^{\infty }\;$ /10^−2^	$D_{w}{\;}^{a}$ /10^−15^ /m^2^ s^−1^	$w_{w}^{\infty }\;$ /10^−2^	$D_{w}{\;}^{b}$ /10^−15^ /m^2^ s^−1^	$w_{w}^{\infty }\;$ /10^−2^	$D_{w}{\;}^{c}$ /10^−15^ /m^2^ s^−1^	$w_{w}^{\infty }\;$ /10^−2^	$D_{w}{\;}^{d}$ /10^−15^ /m^2^ s^−1^
9.24	0.88 ± 0.1	135.0 ± 30	0.47 ± 0.1	46.4 ± 25.1	1.69 ± 0.4	104 ± 55	0.86 ± 0.1	81.4 ± 2.69
29.4	2.99 ± 0.1	64.1 ± 4.15	1.35 ± 0.1	30.5 ± 6.01	5.84 ± 0.4	58.9 ± 3.8	2.46 ± 0.2	27.5 ± 2.89
44.5	4.76 ± 0.1	18.2 ± 2.87	2.07 ± 0.1	14.8 ± 0.634	9.02 ± 0.3	19.1 ± 0.95	3.73 ± 0.2	9.67 ± 2.51
59.9	7.24 ± 0.1	18.1 ± 2.39	3.03 ± 0.2	10.3 ± 0.946	12.8 ± 0.3	29.0 ± 1.82	5.46 ± 0.2	7.17 ± 1.42
73.4	10.4 ± 0.1	41.5 ± 3.48	4.36 ± 0.1	11.4 ± 0.899	17.3 ± 0.3	60.4 ± 3.6	7.82 ± 0.3	7.85 ± 1.62
87.8	17.4 ± 0.2	80.8 ± 0.62	6.86 ± 0.2	30.8 ± 2.52	26.0 ± 0.2	78.9 ± 2.3	12.5 ± 0.5	25.2 ± 6.92

The thickness of the ASD films where ^a^ $L_{0}=8.15\pm 0.22\;µm$ and ^b^ $L_{0}=8.67\pm 0.31\;µm$; ^c^ $L_{0}=8.56\pm 0.28\;µm$, ^d^ $L_{0}=7.65\pm 0.7\;µm$.

## Data Availability

Data is contained within the article or Supplementary Material.

## Supplementary Materials

The following supporting information can be downloaded at: https://www.mdpi.com/article/10.3390/pharmaceutics14061181/s1. Equations (S1)–(S6) Derivation of Equation (13) from the Maxwell–Stefan formalism. Figure S1: Determination of the binary interaction parameter PVPVA and IND. Figure S2: Experimental water-sorption curves in PVP–IND ASDs and fittings to the water-sorption kinetics. Figure S3: Predictions for water-diffusion coefficients in PVP–IND ASDs. References [9,10,20,28,29] are cited in the supplementary materials.
