# Supplementary material for: Predicting the Water Sorption in ASDs
Source: Pharmaceutics. 2022 May 31;14(6):1181. doi: 10.3390/pharmaceutics14061181 (PMC9230848; doi:10.3390/pharmaceutics14061181)
Supplement: Supplementary file 1 [file pharmaceutics-14-01181-s001.zip › pharmaceutics-1742325-supplementary.pdf]

pharmaceutics

Supplementary Material

Predicting the water sorption in ASDs

Dominik Borrmann, Andreas Danzer, and Gabriele Sadowski *

Department of Chemical and Biochemical Engineering, Laboratory of Thermodynamics, TU Dortmund University, Emil-Figge-Str. 70, D-44227 Dortmund, Germany
* Correspondence: gabriele.sadowski@tu-dortmund.de; Tel.: +49-231-755-2635

Derivation of the mixing rule of the water-diffusion coefficient

The initially proposed formulation by Fornasiero et al. [1] was reduced to S1.

$$\frac{\nabla \mu_i}{RT} = \sum_j \frac{w_j}{Đ''_{ij}} r_i \left(u_i - u_j\right) \qquad \text{S1}$$

u_i are the velocities of the component i and w_i are the respective weight fractions. r_i is the mass segments number of component i. Then, $r_i = \frac{M_i}{M_w}$, where M_i is the molar mass of component i and M_w is the molar mass of water which is chosen as the standard for the mass of a segment ($r_w = 1$). The gradient ∇ of the chemical potential μ_i of a component i drives diffusion. We consider one-dimensional isothermal diffusion, and the gradient becomes $\nabla = \left(\frac{\partial}{\partial z}\right)_T$ corresponding to the spatial dimension z perpendicular to its base. The quantity $Đ''_{ij}$ represents a symmetric Maxwell–Stefan diffusion coefficient describing the friction between a segment of i and a segment of j.

Sorption of a water w is considered in an arbitrary number of stationary components $j \neq w$. We apply S1 for $i = w$ (water) which is displayed in S2.

$$\frac{\nabla \mu_w}{RT} = \sum_j \frac{w_j}{Đ''_{wj}} r_w \left(u_w - u_j\right) \qquad \text{S2}$$

The velocities of each component i are related to the mass flux $\dot{m}_i$ of component i via the relation $u_i = \frac{\dot{m}_i}{\rho_i} + u_{ref}$ with u_{ref} being a reference velocity. We assume that the stationary components (e.g., the polymer and the API) have vapor pressure of zero. Then it can be assumed that the mass flux of each component other than water is zero $\dot{m}_j = 0$ which leads to S3.

$$\frac{\nabla \mu_w}{RT} = \frac{\dot{m}_w}{\rho_w} \sum_{j \neq w} \frac{w_j}{Đ''_{wj}} \qquad \text{S3}$$

We expand the right hands-side by the sum of the weight fractions of each component other than the water $1 - w_w$ in S4.

$$\frac{\nabla \mu_w}{RT} = \frac{\dot{m}_w}{\rho_w} (1 - w_w) \sum_{j \neq w} \frac{\frac{w_j}{1 - w_w}}{Đ''_{wj}} \qquad \text{S4}$$

Where one can identify that $\frac{w_j}{1 - w_w} = w_{0j}$, where w_{0j} is the weight fraction of each component in the dry ASD.

Finally, S4 is solved explicitly for $\dot{m}_w$ as shown in S5 with S6.

$$\dot{m}_w = \rho_w \frac{Đ''_w}{1 - w_w} \frac{\nabla \mu_w}{RT} \qquad \text{S5}$$

$$Đ''_w = \left(\sum_{j\neq w} \frac{w_{0j}}{Đ''_{wj}}\right)^{-1} \quad \text{S6}$$

The fractions w_{0j} of the dry ASD do not change and the quantity $Đ''_w$ is a constant. Moreover, the structure of S5 remains the same for any number of stationary components. Thus, the inverse of the water-diffusion coefficient $Đ''_w$ in a mixture of an any number of stationary components is based on the weighted average of the inverse water-diffusion coefficients $Đ''_{wj}$ in the stationary components $j \neq w$.

Determination of the binary interaction between IND and PVPVA

Although the binary interaction parameter between polymer and API had only a neglectable influence on modeling the water-sorption isotherm of the ASDs, it was determined for completeness. As a result, it is not crucial for the proposed methodology of predicting water-sorption isotherms and water-sorption kinetics in ASDs and can also be assumed to be zero with only slight deviations. Here, it was determined by fitting the solubilities of IND in PVPVA at different temperatures. The API weight fraction that fulfills the solid-liquid equilibrium condition in S7 displays its solubility in the ASD.

$$x_a^L = \frac{1}{\gamma_a^L} \exp\left(-\frac{\Delta h_{0a}^{SL}}{RT}\left(1 - \frac{T}{T_{0a}^{SL}}\right) + \frac{\Delta c_p^{SL}}{RT}\left(\frac{T_{0a}^{SL}}{T} - 1 - ln\left(\frac{T_{0a}^{SL}}{T}\right)\right)\right) \quad \text{S7}$$

Here, x_a^L is the solubility of the API in the ASD and γ_a^L is the activity coefficient of API calculated from PC-SAFT. T is the temperature, Δh_{0a}^{SL} is the melting enthalpy of the API, T_{0a}^{SL} is the melting temperature of the API, and Δc_p^{SL} is the heat capacity difference between amorphous and solid API. The melting properties of IND were taken from Paus et al. [2] ($\Delta h_{0a}^{SL} = 39.3 \frac{kJ}{mol}$, $\Delta cp^{SL} = 116.95 \frac{J}{mol\,K}$, $T_{0a}^{SL} = 433.25\ K$). The fitting to the solubility of IND in PVPVA from Prudic et al. [3] is displayed in Fig. S1.

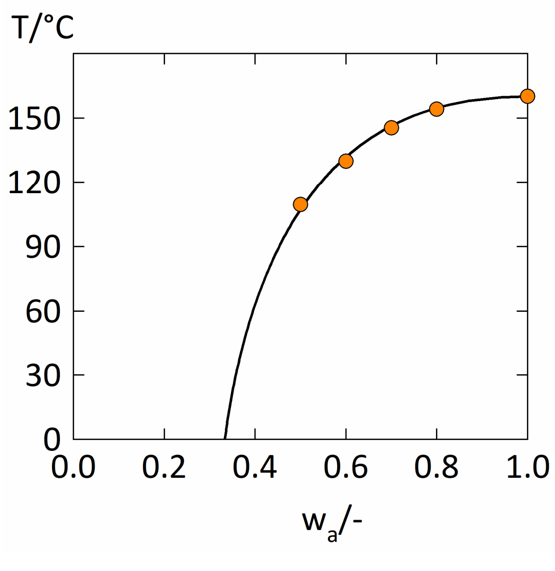

Figure S1: The solubilities of IND in PVPVA taken from Prudic et al. [3] were displayed as circles. The fitting to S7 is displayed as a solid line.

PC-SAFT accurately represents the solubility of IND in PVPVA while considering PVPVA as a homopolymer using a constant temperature-independent binary interaction parameter $k_{ij} = -0.0621$.

Experimental water-sorption curves

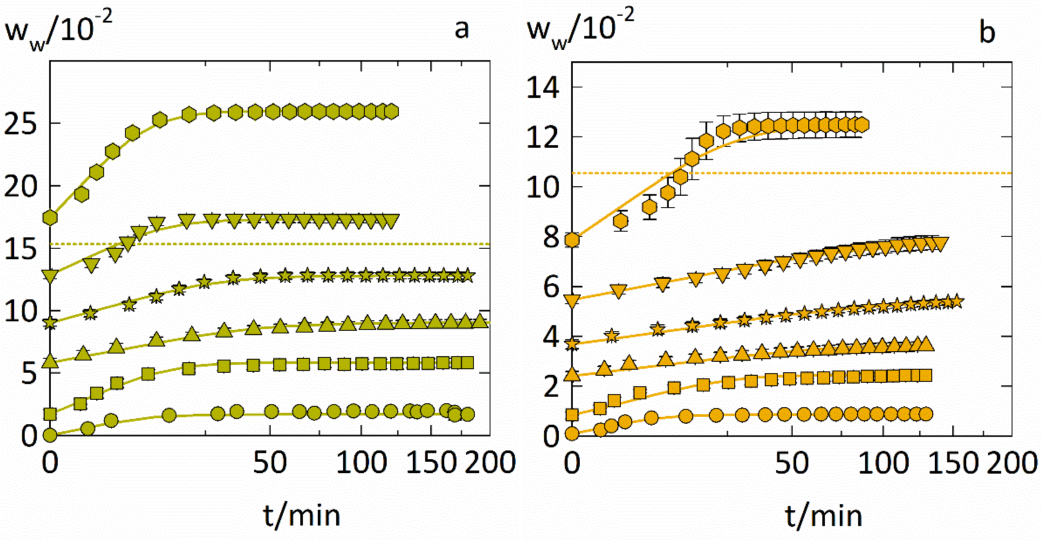

Figure S2: Water-sorption curves in PVP–IND ASD at T=25°C. The evolution of the water-mass fractions in ASDs with drug loads of 0.2 (a) and 0.5 (b) are displayed for six RH step changes. Each step change is displayed via different symbols (circles: 0 to 0.1 RH, squares: 0.1 to 0.3 RH, up-side triangles: 0.3 to 0.45 RH, stars: 0.45 to 0.6 RH, down-side triangles: 0.6 to 0.75 RH, hexagons: 0.75 to 0.9 RH) while the fittings with Equation (10) are indicated as solid lines. Predictions of the water-weight fraction that result in a T_g of 25°C by Equation (15) is displayed as dotted horizontal lines.

Prediction of water-diffusion coefficients in ASDs

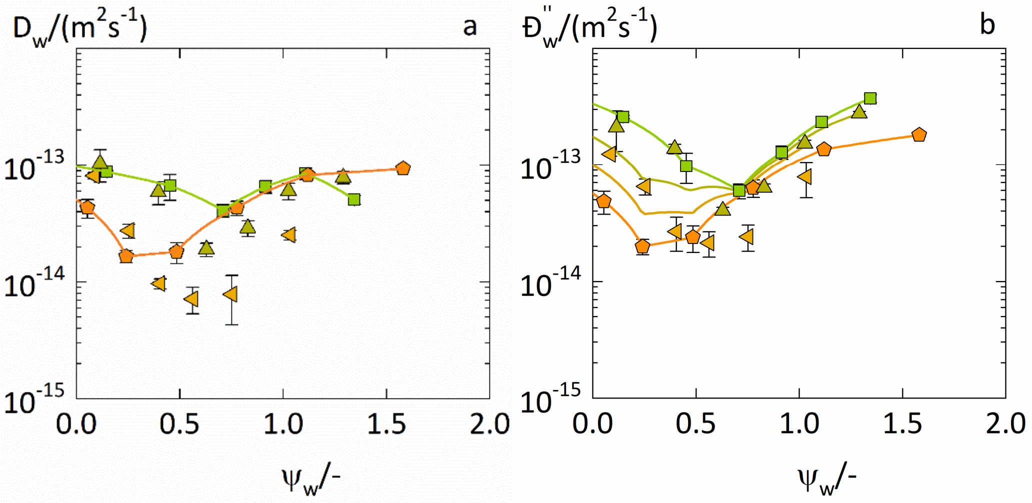

Figure S3: Water-diffusion coefficients $Đ''_w$ and D_w in PVP–IND ASDs at 25°C. Fickian water-diffusion coefficients D_w in ASDs (a) from fittings of Equation (10) and the corresponding segmental Maxwell–Stefan diffusion coefficients $Đ''_w$ in ASDs (b) via Equation (11) are displayed as up-side triangles (for a drug load of 0.2) and left-side triangles (drug load of 0.5), respectively. The Fickian diffusion coefficients D_{wa} of water in IND (pentagons) and D_{wp} of water in PVP (squares) were taken from previous works [4,5]. Additionally, the predicted Maxwell–Stefan diffusion coefficients $Đ''_w$ of water in the ASDs are displayed as solid lines.

References

1. Fornasiero, F.; Prausnitz, J.M.; Radke, C.J. Multicomponent Diffusion in Highly Asymmetric Systems. An Extended Maxwell–Stefan Model for Starkly Different-Sized, Segment-Accessible Chain Molecules. *Macromolecules* **2005**, *38*, 1364–1370, doi:10.1021/ma040133v.

2. Paus, R.; Ji, Y.; Braak, F.; Sadowski, G. Dissolution of Crystalline Pharmaceuticals: Experimental Investigation and Thermodynamic Modeling. *Ind. Eng. Chem. Res.* **2015**, *54*, 731–742, doi:10.1021/ie503939w.

3. Prudic, A.; Kleetz, T.; Korf, M.; Ji, Y.; Sadowski, G. Influence of Copolymer Composition on the Phase Behavior of Solid Dispersions. *Mol. Pharm.* **2014**, *11*, 4189–4198, doi:10.1021/mp500412d.

4. Borrmann, D.; Danzer, A.; Sadowski, G. Water Sorption in Glassy Polyvinylpyrrolidone-Based Polymers. *Membranes (Basel).* **2022**, *12*, 434, doi:10.3390/membranes12040434.

5. Borrmann, D.; Danzer, A.; Sadowski, G. Measuring and Modeling Water Sorption in Amorphous Indomethacin and Ritonavir. *Mol. Pharm.* **2022**, *19*, 998–1007, doi:10.1021/acs.molpharmaceut.1c00984.
